# Psychosocial distress under pressure

**DOI:** 10.1007/s12471-013-0508-6

**Published:** 2014-01-08

**Authors:** C. A. Swenne

**Affiliations:** Cardiology Department, Leiden University Medical Center, POB 9600, 2300 RC Leiden, the Netherlands

In the current issue of the Netherlands Heart Journal, Ringoir and colleagues report an interesting observation: in a cohort of 605 elderly (60–85 years) primary care patients, with hypertension but without heart failure or current treatment by a cardiologist, no significant association between symptoms of psychological distress (depression, anxiety and type D personality) and systolic blood pressure > 160 mmHg could be demonstrated [1].

Possibly, this study is underpowered and a larger study group would have shown a significant association for type D personality (the reported adjusted odds ratio for type D personality was 1.563 with a confidence interval of 0.805–3.038). Possibly, this study suffers from selection bias (only 68 % of the invited patients agreed to participate, and personality plays a role in the decision to participate in research studies or not [2, 3].

These, and other limitations reported by the authors, make it difficult to draw definite conclusions and leave us with question marks to be added to the multiple existing question marks regarding the role of personality characteristics and psychosocial factors in the development and progression of cardiovascular disease, and the options for successful intervention in this respect. At various instances in the Introduction and the Discussion, the authors position their rationale and findings amidst this ongoing discussion.

It is, indeed, striking that the published odds ratios of the association between type D personality and mortality/nonfatal myocardial infarction have decreased over time [4], a result that should most likely be ascribed to methodological differences between earlier and later studies. The most recent European Society of Cardiology guidelines on cardiovascular disease prevention in clinical practice [5] endorse on pages 1653–1655 and 1671–1672 the view that psychosocial factors, including type D personality, contribute both to the risk of developing cardiovascular disease and to the worsening of the clinical course and prognosis of cardiovascular disease, amongst others because these factors act as barriers to treatment adherence and efforts to improve lifestyle, as well as to promoting health and wellbeing in patients and populations. On the other hand, effects of intervention with these psychosocial factors are as yet not really clear; e.g., the guidelines conclude that evidence that treatment of clinically significant depression and anxiety will improve cardiac endpoints is inconclusive.

Beyond any doubt, further research, preferably by multidisciplinary research groups, is needed to make clear if and to what extent psychosocial intervention could help to decrease the burden of cardiovascular disease.
